# Differences in synovial fluid cytokine levels but not in synovial tissue cell infiltrate between anti-citrullinated peptide/protein antibody-positive and –negative rheumatoid arthritis patients

**DOI:** 10.1186/ar4372

**Published:** 2013-11-07

**Authors:** José A Gómez-Puerta, Raquel Celis, M Victoria Hernández, Virginia Ruiz-Esquide, Julio Ramírez, Isabel Haro, Juan D Cañete, Raimon Sanmartí

**Affiliations:** 1 Arthritis Unit, Department of Rheumatology, Hospital Clinic, Barcelona, Spain; 2 Unit of Synthesis and Biomedical Applications of Peptides, IQAC-CSIC, Jordi Girona, Barcelona, Spain

## Abstract

**Introduction:**

Comparative data on synovial cell infiltrate and cytokine levels in anti citrullinated peptide/protein antibody (ACPA)-positive and ACPA negative rheumatoid arthritis (RA) patients are scarce. Our aim was to analyze synovial cell infiltrate and synovial fluid (SF) levels of cytokines in patients with RA according to the presence or absence of ACPA in serum.

**Methods:**

A cross-sectional study in a single center including consecutive RA patients was performed. Patients were defined as 'ACPA negative' if serum was negative to two different ACPAs [second generation commercial anti-cyclic citrullinated peptide antibodies (CCP2) and chimeric fibrin/filaggrin citrullinated antibodies]. Parallel synovial tissue (ST) biopsies and SF were obtained by knee arthroscopy. Synovial cell infiltrate and endothelial cells were analyzed by immunohistochemistry and SF levels of Th1, Th2, Th17 and pro-inflammatory cytokines by Quantibody(R) Human Array.

**Results:**

A total of 83 patients underwent arthroscopy, with a mean age of 55.9 ± 12 years, and mean disease duration of 45 months (interquartile range, IQR 10.8 to 122). 62% were female and 77% were ACPA positive. No significant differences were found in clinical variables, acute phase reactants, synovial cell infiltrate or lymphoid neogenesis (LN) between ACPA positive and negative patients. However ACPA positive patients had significantly higher levels of IL-1β, IL-10, IL-17 F and CC chemokine ligand 20 (CCL-20) than ACPA negative patients.

**Conclusions:**

In our cohort of patients with RA no significant differences were found in synovial cell infiltrate or synovial LN according to ACPA status. However, ACPA positive patients had higher levels of T-cell derived and pro-inflammatory cytokines than ACPA negative patients. As systemic and local inflammation was similar in the two groups, these findings support a distinct synovial physiopathology.

## Introduction

Rheumatoid arthritis (RA) is a heterogeneous chronic inflammatory disease conditioned by genetic, environmental and immunological factors, mainly autoantibodies. There is evidence that RA patients positive for rheumatoid factor (RF) and anti-citrullinated peptide/protein antibodies (ACPA) have more severe disease and poorer outcomes [1]. ACPA-positive RA patients develop earlier and more widespread erosions than consistently ACPA-negative patients [1]. It has been suggested that although early clinical phases could be similar in ACPA positive and negative patients, ACPA positive RA patients develop more structural damage [2] and more cardiovascular disease during follow-up [3].

Different peptides/proteins present in the synovial membrane have been proposed as *in vivo* antigens for anti-citrullinated antibodies, including mutated vimentin [4], fibrinogen [5] and α-enolase [6]. Different citrullinated antigenic substrates have been developed in order to improve the sensitivity and specificity of ACPA tests for RA diagnosis, showing a good but not absolute correlation between them [7]. Our group demonstrated that a homemade ACPA ELISA test, using chimeric fibrin/filaggrin citrullinated synthetic peptides (CFFCP) [8] had a high sensitivity and specificity for RA, and was able to detect some patients negative to commercial anti-cyclic citrullinated peptide antibodies (CCP2), the most common test used in clinical practice. Additionally, early RA patients who were positive for anti-CFFCP had a poor radiological outcome [9].

Despite the body of knowledge of the different clinical phenotype of RA patients according to ACPA status, there is limited data on their synovial fluid (SF) cytokine levels and the characteristics of synovial membrane infiltrates [10-12]. Given that differences in synovial cell infiltrate between RA and spondyloarthropathy (SpA) have been described, with significantly more mast cells [13], neutrophils (innate immunity) and endothelial cells in SpA [14], the objective of this study was to analyze whether these cells are also overrepresented in ACPA negative RA patients, and whether cells related to adaptive immunity (T and B cells and lymphoid neogenesis structures) are more abundant in ACPA positive patients. We also analyzed T-cell derived and pro-inflammatory cytokine levels in SF of RA patients according to ACPA status.

## Methods

We conducted a cross-sectional study in a single center that included patients undergoing rheumatologic arthroscopy from a cohort of 300 consecutive RA patients attending the Rheumatology Department, Hospital Clinic, Barcelona, Spain, a tertiary referral center. Patients with long-standing disease (>20 years of evolution) were excluded. All patients gave informed consent, and the study was approved by the Ethics Committee of the Hospital Clinic of Barcelona, Barcelona, Spain.

### Selection of sample

All patients fulfilled American College of Rheumatology (ACR) 1987 classification criteria [15] according to clinical judgment at diagnosis. Data were recorded using a specifically-created standard data collection form and included detailed demographic, clinical and laboratory variables. RF was determined by nephelometry, CCP2 antibodies by ELISA (Eurodiagnostica) and anti-CFFCP by a home-made ELISA, as previously described [9].

RA patients negative for both ACPA antibody tests were classified as seronegative and patients positive at least for one of the antibodies (CCP2 or CFFCP) before arthroscopy as seropositive.

Patients who fulfilled criteria for another inflammatory arthropathy during follow-up, mainly psoriatic arthritis (PsA) (CASPAR critera) [16], SpA (European Spondyloarthropathy Study Group) [17] or systemic lupus erythematosus (ACR criteria 1997) were excluded [18].

Synovial samples were obtained by knee arthroscopy. The indication for diagnostic (exclusion of other causes of knee arthritis) or therapeutic (lavage with four liters of saline and glucocorticoid infiltration) arthoscopy was made by clinical judgment in patients with an actively inflamed knee, defined as a swollen joint with inflammatory SF. Arthroscopy was performed with a 2.7 mm arthroscope (Storz, Tuttlingen, Germany) under local anesthesia.

### Immunohistochemistry of synovial tissue

Synovial biopsies were fixed in 10% neutral formalin and embedded in paraffin, sectioned, and subjected to antigen retrieval by microwave heating in 1 mM ethylenediaminetetraacetic acid (EDTA) for 15 minutes when required. The slides were subsequently stained with an automated immunostainer (TechMate 500 Plus; Dako, Cambridge, UK) using the following monoclonal antibodies: anti-CD3 (clone PS1; Novocastra, Newcastle, UK), anti-CD20 (clone L26; Dako), anti-CD15 (clone BY87; Novocastra), anti-CD31 (clone JC70A; Dako), anti-CD68 (clone KP-1; Dako), anti-CD117 (mast cells, rabbit anti-human polyclonal antibody; Dako).

As a negative control, primary antibodies were substituted by non-immune matched immunoglobulins. Primary antibodies were subsequently detected by an avidin-biotin-peroxidase-based method (Envision System; Dako) and an aminoethylcarbazole color reaction (Sigma-Aldrich, St. Louis, MO, USA), as described previously in detail [19]. Finally, the slides were counterstained with hematoxylin.

Lymphocytes (CD3, CD20), neutrophils (CD15), macrophages (CD68), mast-cells (CD117) and vessels (endothelial cells, CD31) were quantified by Digital Image Analysis (Olympus®, Tokyo, Japan) by an expert (RC) blinded to the clinical data [19]. We also evaluated the presence of lymphoid neogenesis (LN) as defined by the presence of follicular aggregates grade ≥2 (>6 radial B-cells) with T/B cell segregation and high endothelial venules (that is, MECA-79 epitope positive) [20]. We have previously demonstrated that this definition correlates well with synovial tissue (ST) expression of LN-associated chemokines [21].

### Cytokine analysis in synovial fluid

SF concentrations (pg/ml) of Th1, Th2, Th17 and pro-inflammatory cytokines were determined by Quantibody® Human Th17 Array (RayBiotech, Norcross, GA, USA) and included Granulocyte macrophage-colony stimulating factor, (GM-CSF), IFNγ, -1β, IL-2, IL-4, IL-5, IL-6, IL-10, IL-12p70, IL-13, IL-17, IL-17 F, IL-22, IL-23, CCL 20, transforming growth factor (TGF)-β1, TNFα, and TNFβ. Quantibody (R) is an array-based multiplex ELISA system for simultaneous measurements of multiple cytokines [22].

### Statistical analysis

Several variables were dichotomized for the analysis. Patients were labeled as seronegative if they were negative for both antibodies, as described above. Patients were compared according to ACPA status. For immunohistochemistry analysis, we first stratified the cohort in two categories (ACPA positive and APCA negative), but we also made a sensitivity analysis dividing the cohort in two subcategories: ACPA negative and ACPA positive at high titers, using the CCP2 test (>1,600 IU). Differences in means in normally distributed variables were analyzed using the parametric *T*-test, and variables without a normal distribution were assessed using Wilcoxon’s test. Correlations were assessed using either the Pearson correlation test for normally distributed data or Spearman’s *P* when data were not normally distributed. Multiple regression models were used for disease activity and disease duration adjustments. The analysis was made using the IBM SPSS v20 statistical package.

## Results

The study included 83 patients with RA who underwent rheumatologic arthroscopy, with a mean age at arthroscopy of 55.9 ± 12 years, and mean disease duration of 45 months (interquartile range (IQR) 10.8 to 122 months). Of the 83 patients, 62% were women, 88% were white, 74% had erosive disease and 31% extra-articular disease. A total of 26 (31%) were receiving biological treatment. RF was positive in 65 (78%) and ACPA in 64 (77%) patients; nine patients were positive for anti-CFFCP and negative for anti-CCP2, while four patients were positive for CCP2 but negative for anti-CFFCP. Forty-four patients were positive for both antibodies.

No clinically important differences were found between ACPA positive and ACPA negative patients. Both groups had a similar profile in terms of disease duration, clinical disease activity, remission rates, biological activity (erythrocyte sedimentation rate (ESR) and C-reactive protein (CRP) levels), erosive disease, disability and treatment (Table 1). As expected, RF was more prevalent in ACPA positive patients (87% versus 47%, *P* = <0.001).

**Table 1:** Clinical characteristics of 83 patients with arthroscopy

**Variables**	**Total**	**ACPA positive**	**ACPA negative**	***P *** **value**
	**Number = 83**	**Number = 64**	**Number = 19**	
Sex (Female%)	62	64	58	0.62
Age at arthroscopy (Mean, SD)	55.98 ± 12.7	55.29 ± 12.8	58.23 ± 12.3	0.47
Disease duration to arthroscopy (Median, IQR 25 to 75) months	45.50 (10.8–122.1)	34.56 (8.8–122.0)	79.0 (15.1–126.8)	0.27
Rheumatoid factor (%)	78	87	47	<0.001*
Extra-articular disease (%)	31	35	20	0.28
Erosive disease (%)	74	72	76	0.73
CRP mg/dl (Median, IQR 25 to 75)	2.32 (1.0–5.3)	2.95 (1.0–5.9)	1.43 (0.7–3.7)	0.21
ESR mm (Median, IQR 25 to 75)	32 (16.0– 64.0)	37 (17.5–70.0)	26 (14.7–39)	0.07
DAS28 (Median, IQR 25 to 75)	4.74 (3.6–5.5)	4.81 (3.7–5.5)	4.74 (3.2–5.5)	0.52
mHAQ (Median, IQR 25 to 75)	1.19 (0.5–1.5)	1.25 (0.6–1.6)	0.56 (0.2–0.7)	0.10
Number of DMARDs (ever), Mean (SD)	2.20 (1.1)	2.20 (1.1)	1.86 (1.1)	0.89
Methotrexate (%), ever	88.4	88.9	88.7	0.98
Biologic treatment (%, before arthroscopy) ever	40.6	40.0	42.0	0.85

ACPA, anti citrullinated peptide/protein antibodies; CRP, C-reactive protein; DAS-28, disease activity score -28 joints; DMARDs, disease-modifying anti-rheumatic drugs; ESR, erythrocyte sedimentation rate; IQR, interquartile range; mHAQ, Modified Health Assessment Questionnaire, SD, standard deviation; SJC, swelling joint count; TJC, tender joint count. *p value is significant if <0.05.

### Synovial tissue infiltrate and lymphoid neogenesis are similar between ACPA negative and positive rheumatoid arthritis patients

**Figure 1:**
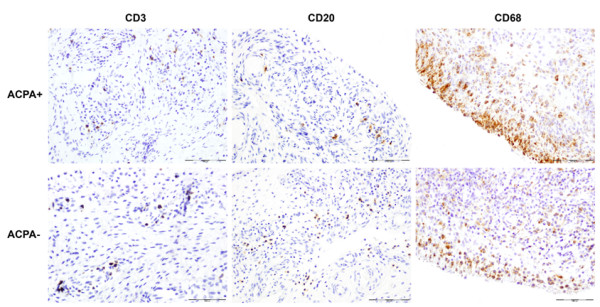
**Microscopic analysis of synovial inflammation with immunostaining for T-cells (CD3+), B-cells (CD20+) and macrophages (CD68+) in ACPA positive (+) versus ACPA negative (-) RA.** Original magnification × 20. ACPA, anti-citrullinated peptide/protein antibodies; RA, rheumatoid arthritis.

When the immunohistochemical analysis of synovial membrane was compared according to ACPA status (positive versus negative), including CD3 (T-cell), CD20 (B-cell), CD15 (neutrophils), CD68 (macrophages) in lining and sublining, CD117 (mast cells) and vessels (CD31, endothelial cells), there were no significant differences between the two groups (Figure 1 and Table 2). LN was more prevalent in ACPA negative patients, but without significant differences (58.8% versus 39.5%, = 0.119). Stratification of patients into ACPA positive at the highest titers (CCP2 >1,600 IU) and ACPA negative showed no significant differences in ST infiltrates. Once again, LN was non-significantly more prevalent in ACPA negative patients (58.8% versus 25%, = 0.71) (Additional file 1: Table S1).

**Table 2:** Immunohistochemistry characteristics according to ACPA status

**Synovial cells**	**Total number = 83**	**ACPA positive number = 64**	**ACPA negative number = 19**	***P *** **value**
CD3/mm^2^ (Median, IQR 25 to 75)	509.90(210.7–821.6) n = 70	514.10 (198.9–821.6) N = 54	509.30 (233.8–898.2) n = 16	0.823
CD20/mm^2^ (Median, IQR 25 to 75)	126.52 (30.3–242.6) n = 70	117.90 (26.6–235.4) n = 54	162.73 (42.2–315) n = 16	0.386
CD20 cell grade 3 follicles (Median, IQR 25 to 75)	0 (0–2.7) n = 72	0 (0–2) n = 55	2.0 (0–3.5) n = 17	0.221
CD68L/mm^2^ (Median, IQR 25 to 75)	293.34 (94.2–453.4) n = 63	305.59 (94.2–438.5) n = 47	204.74 (93.5–532.2) n = 16	0.813
CD68SL/mm^2^ (Median, IQR 25 to 75)	677.17 (3.1–1665.5) n = 63	738.50 (379.3–2016.7) n = 47	526.74 (271.5–874.9) n = 16	0.138
CD15/mm^2^ (Median, IQR 25 to 75)	58.46 (10–204.4) n = 49	71.90 (9.0–445.4) n = 35	35.74 (9.9–88) n = 14	0.278
CD117/mm^2^ (Median, IQR 25 to 75)	40.26 (20.8–55) n = 51	39.27 (21.2–54.5) n = 37	42.81 (19.1–75.6) n = 14	0.473
CD31/34 (Median, IQR 25 to 75)	46.31 (37.4–64.1) n = 44	53.66 (36.8–68.8) n = 31	43.56 (34–63) n = 13	0.425
LN (%)	40.80	39.50	58.80	0.119

ACPA, anti-citrullinated peptide/protein antibodies; IQR, interquatile range; LN, lymphoid neogenesis; n, number.

We also evaluated the immunohistochemical findings adjusted by disease activity and disease duration, but no differences in ST infiltrate were found according to these factors in the multivariate regression model (data not shown).

### ACPA positive rheumatoid arthritis patients have higher levels of IL-17 F, CCL20, IL-1β and IL-10 in synovial fluid

**Figure 2:**
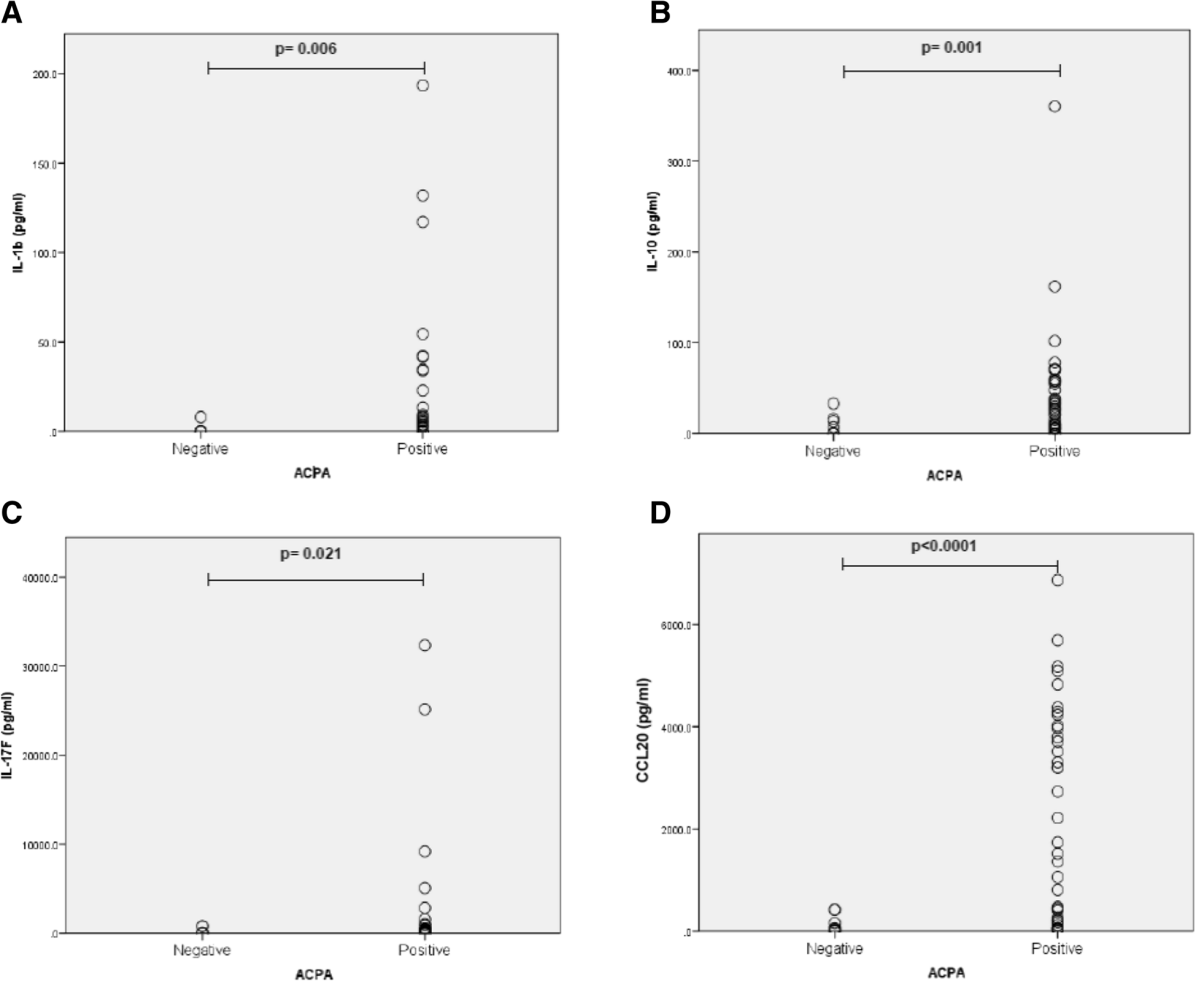
**Differential expression of cytokines in SF ACPA positive and negative patients.** (**A.** IL-1β, **B.** IL-10, **C.** IL-17 F, **D.** CCL20). SF concentrations (pg/ml) were determined by Quantibody® Human Th17 Array (RayBiotech, Norcross GA, USA). Wilcoxon’s test was used to compare means in **A-D**. ACPA, anti-citrullinated peptide/protein antibodies; SF, synovial fluid.

SF analysis was available for 51 patients (40 ACPA positive/11 negative). Except for the expected higher RF positivity, there were no significant clinical differences between the two groups (Table 3). ACPA positive patients had significantly higher levels of IL-17 F, CCL20, IL-1β and IL-10 than ACPA negative patients (Figure 2). No differences in other cytokines, including IL-6 and tumor necrosis factor alpha (TNFα), were found (Table 4).

**Table 3:** Comparison of patients with SF cytokine analysis

**Variables**	**Total number = 51**	**ACPA positive number = 40**	**ACPA negative N = 11**	***P *** **value**
**Sex (female,%)**	64.7	65.0	63.6	0.93
**Age at arthroscopy, years (Mean, SD)**	56.8 ± 12.1	58.5 ± 12.2	58.3 ± 1mber2.2	0.64
**Disease duration to arthroscopy (months), Median (IQR 25–75)**	52.0(12.2–134.3) N = 47	39.3 (8.7–134.3) N = 39	87.9(45.7–147.4) N = 8	0.32
**Rheumatoid factor (%)**	82	90	54	0.006*
**Erosive disease (%)**	72	72	70	0.87
**Extra-articular disease (%)**	37	40	25	0.41
**DAS28, Median (IQR 25–75)**	5.06 (3.9–5.8) N = 47	5.06 (4.0–5.7) N = 37	5.13 (3.8–5.9) N = 10	0.81
**ESR mm/hr (before arthroscopy), Median (IQR 25–75)**	43 (22–70) N = 47	43 (24–71) N = 37	27 (16–66) N = 10	0.42
**CRP mg/dl (before arthroscopy), Median (IQR 25–75)**	3.0 (1.2–6.2) N = 47	3.1 (1.1–6.6) N = 37	2.2 (1.1–6.3) N = 10	0.82
**DMARDs (Mean, SD)**	2.61 ± 1.50	2.43 ± 1.48	3.27 ± 1.60	0.10
**Biologic treatment (%) ever**	49	40	82	0.014*

Data are expressed as median (IQR) or as percentage. CRP, C-reactive protein; DMARDs, disease modifying anti-rheumatic drugs; ESR, erythrocyte sedimentation rate; mHAQ, Modified Health Assessment Questionnaire; IQR, interquartile range; SF, synovial fluid; SJC, swelling joint count; TJC, tender joint count. * p value is significant if < 0.05.

**Table 4:** Comparison of cytokine profile in SF according to ACPA status (number = 51)

**SF cytokines**	**Total**	**ACPA positive**	**ACPA negative**	***P *** **value**
	**Number = 51**	**Number = 40**	**Number = 11**	
GM-CSF (pg/ml)	0 (0 to 0)	0 (0 to 0)	0 (0 to 0)	0.461
IFNγ (pg/ml)	0 (0 to 0)	0 (0 to 0)	0 (0 to 0)	0.828
IL-1β (pg/ml)	0 (0 to 7.9)	2.61 (0 to 12.2)	0 (0 to 0)	**0.011**
IL-2 (pg/ml)	0 (0 to 159.3)	38.9 (0 to 164)	0 (0 to 0)	0.076
IL-4 (pg/ml)	0 (0 to 0)	0 (0 to 0)	0 (0 to 0)	0.280
IL-5 (pg/ml)	0 (0 to 0)	0 (0 to 0)	0 (0 to 0)	0.714
IL-6 (pg/ml)	1,252.6 (4.3 to 47.4)	1,260.8 (774.9 to 1,725.1)	1,170.6 (628.8 to 1,568.4)	0.697
IL-10 (pg/ml)	16.2 (4.3 to 47.4)	26.7 (6.9 to 57.1)	0 (0 to 13.4)	**0.001**
IL-12p70 (pg/ml)	0 (0 to 0)	0 (0 to 0)	0 (0 to 0)	0.682
IL-13 (pg/ml)	0 (0 to 0)	0 (0 to 0)	0 (0 to 0)	0.688
IL-17 (pg/ml)	0 (0 to 26.2)	0 (0 to 33.2)	0 (0 to 0)	0.160
IL-17 F (pg/ml)	142.7 (0 to 532.6)	269.9 (23.1 to 534.8)	9.2 (0 to 71.2)	**0.021**
IL-21 (pg/ml)	783 (0 to 1,162.4)	820.8 (0 to 1,158.3)	0 (0 to 1,381.4)	0.493
IL-22 (pg/ml)	0 (0 to 0)	0 (0 to 91.8)	0 (0 to 0)	0.207
IL-23 (pg/ml)	259 (0 to 863.1)	311.7 (0 to 1,028.4)	169.4 (0 to 472.3)	0.372
CCL20 (pg/ml)	445.5 (59.0 to 3,521.1)	1,437.9 (179.3 to 3,928.0)	39.2 (5.6 to 161.9)	**<0.0001**
TGF-β1 (pg/ml)	768 (0 to 1,927.7)	924.4 (0 to 1,918.3)	0 (0 to 2,035.3)	0.277
TNFα (pg/ml)	0 (0 to 16.3)	0 (0 to 16.2)	0 (0 to 17.6)	0.636
TNFβ (pg/ml)	0 (0 to 13.1)	0 (0 to 13)	0 (0 to 15.2)	0.759

Data are expressed as median (IQR). CCL20: CC chemokine ligand 20, GM-CSF: Granulyte macrophage-colony stimulating factor, TGF: Transforming growth factor, TNF: Tumor necrosis factor.

There were weak significant correlations between SF CCL20 and disease activity as measured by the disease activity score in 28 joints (DAS28) at baseline (*r* = 0.32; *P* = 0.028, respectively). SF CCL20 levels also significantly correlated with CD-15+ cell count (neutrophils/mm2) (r = 0.42; = 0.011) in ST. No other correlations were found.

## Discussion

We hypothesized that ACPA positive and negative patients might exhibit differences in ST cellularity with respect to innate and adaptive immune cells and T-cell derived cytokine levels. We found higher SF levels of IL-1β, IL-10, IL-17 F and the CCL20 chemokine in ACPA positive patients in comparison with ACPA negative patients. However, we found no significant differences in synovial cell infiltrates or in LN according to ACPA status. These results are underlined by the fact that the two groups were very similar in terms of biological and clinical disease activity. Additionally, the results did not vary after adjustment of cell infiltrate in ST and cytokines levels in SF according to disease activity and disease duration.

Previous studies have reported that ACPA positive and negative RA patients have different susceptibility genes and a different disease course [23]. The HLA shared epitope and PTPN22 [24] predispose to ACPA-positive RA, whereas HLA-DRB1*03 and IRF5 [25] predispose to ACPA-negative disease.

Few studies have focused on synovial differences in RA patients according to ACPA status [10-12]. As in the present study, Cantaert *et al*. [10] found no significant differences in cell infiltrates in ST from 54 RA patients analyzed (37 ACPA positive and 17 ACPA negative).

In contrast, van Oosterhout *et al*. [11] found a higher number of infiltrating lymphocytes and higher expression of CD3, CD8, CXCL12 and CD45RO in 34 ACPA positive patients in comparison with 23 ACPA negative patients, suggesting that ACPA is associated with a higher number of cells from the acquired immune system. The differences persisted after correction for DAS28. Conversely, ACPA negative patients had more extensive signs of fibrosis [11], a rather non-specific finding generally associated with longer disease duration and residual synovitis.

Mast cells have recently been shown to be the main producers of IL-17 in ST in RA [26] and SpA [13], although they are significantly more abundant and produce more IL-17 in ST of SpA than in RA [13]. Neutrophils also produce IL-17 and are over-represented in ST of SpA compared with RA [27]. Our results show that mast cells, neutrophils and macrophages, cells from the innate immune system, are similarly represented in ST of ACPA positive and negative RA patients. This finding is in line with a recent report by Suurmond *et al*. [12] that found no significant differences in CD117+ mast cells, CD3+ lymphocytes and CD68 macrophages in ST of ACPA positive and negative RA patients.

We have previously shown that seronegative RA (RF negative) patients predominantly exhibited a synovial vascular pattern characterized by tortuous vessels [28] similar to that found in patients with SpA [29]. Furthermore, these patients had a higher density of synovial vessels, as determined by CD31, and less radiographic damage than seropositive (RF positive) patients. However, in the present study we found no differences in synovial vessel density or radiographic damage. A possible explanation is that our present cohort of ACPA negative patients may represent some selection bias to a more aggressive disease than usual in this type of patients, as demonstrated by the same level of disease activity, frequency of erosive disease and the need for DMARDs and biological therapy as ACPA positive patients.

Synovial LN, as previously defined, might act as an ectopic lymphoid organ [30]. Recently, our group analyzed the clinical significance of LN in 86 patients with RA [19]. LN was found in around 50% of patients, and was associated with longer disease duration and greater use of anti-TNF therapy. LN was not correlated with other clinical characteristics such as disease activity or RF or ACPA antibodies. After multivariate logistic regression, LN was an independent predictor of a worse response to therapy. In the present study we found no association between synovial LN and ACPA status, confirming the results of previous studies [19,31,32]. Furthermore, we found a slightly higher prevalence of LN in ACPA negative patients, as have other studies [32]. These results are not unexpected, because synovial LN has also been described in around 40% of patients with PsA, a disease with no specific autoantibodies [21]. Therefore, although experimental models suggest that ACPA are produced by plasma cells associated with germinal centers in RA ST [33], clinical studies have failed until now to demonstrate an association between synovial LN and the presence or absence of ACPA in serum or SF (28). Thus, the role of synovial LN in the pathogenesis of RA remains to be elucidated. We recently reported data supporting the idea that synovial LN in PsA could be associated with a different pattern of cytokines that are related to the IL-17/IL-23 pathway [34].

Although our results showed no significant differences in synovial LN between ACPA positive and negative RA patients, we found higher levels of cytokines (IL-1, IL-10, IL-17 F) and the CCL20 chemokine in SF of ACPA positive patients. IL-1 has a clear pro-inflammatory role in RA through stimulation of multiple inflammatory mediators, including cytokines, chemokines and metalloproteases. IL-10 seems to have a more complex functional role, acting as an anti-inflammatory cytokine, but may also stimulate the humoral response and, thus, lead to autoantibody production. In fact, IL-10 may also have pro-inflammatory actions, as shown by reports of an association between IL-10 levels and radiological progression in RA [35,36].

IL-17 F is an isoform of IL-17A and has similar biological effects, with a key role in neutrophilic inflammation. Although Th17 cells originally appeared to be the greatest producers of IL-17, recent studies have shown the scarcity of T cells producing IL-17 in RA synovitis, with mast cells and neutrophils being the main source of synovial IL-17 [26].

SF levels of CCL20, a chemokine ligand for CCR6+ cells, included Th17 cells, correlated with disease activity and were also significantly higher in ACPA positive patients. Furthermore, SF CCL20 levels correlated with neutrophilic infiltration in ST. All these results are in line with previous findings by Melis *et al*. [37], and confirm recent work reporting that ACPA positive RA patients had significantly higher levels of IL-17 in SF compared with ACPA negative patients [12], suggesting that the IL-17 pathway may play a greater role in the pathogenesis of ACPA positive RA.

Our study has several strengths and limitations. First, we defined ACPA status using two different techniques, which allowed more accurate classification, that is, we classified patients as seronegative only when they were negative for both antibodies. We also analyzed patients according to ACPA at high titers versus ACPA negative. Secondly, the study sample was well balanced, without significant differences in terms of clinical characteristics, suggesting that our results reflect phenomena occurring in SF and the synovial membrane that are probably not influenced by disease duration or disease activity. Thirdly, we made a broad synovial assessment, analyzing not only various lines of cell infiltrates and LN in order to compare the relevance of innate or acquired immune cells in each patient group, but also the cytokines associated with Th1, Th2, and Th17 cells. To our knowledge, this is the first study to determine such a broad spectrum of SF cytokines according to ACPA status. A few studies have analyzed a smaller number of cytokines (IL-17, IL-15 or IL-33) [12,38,39] according to ACPA status. Serum levels of IL-33 were significantly higher in patients with high ACPA concentrations [39], whereas serum IL-17 [12] and IL-15 [38] levels were slightly, but non-significantly, higher in ACPA positive patients.

We included one of the largest cohorts of patients with parallel ST and SF samples. However, there were a limited number of ACPA negative patients, even though the proportion reflected well the clinical reality in our setting (around 25%). Likewise, as our hospital is a reference center for inflammatory arthritis, our patients could be a selected sample of RA patients. However, we found no significant differences in our cohort compared with other cohorts comprising mainly white patients [40]. In addition, there was no systematic protocol to determine the indication for arthroscopy. The study was conducted in daily clinical practice and the indication for arthroscopy was made according to clinical judgment. It is interesting to note that although the different clinical characteristics including the level of disease activity were similar between ACPA positive and negative patients, biologic use was more frequent in the latter group, therefore a pharmacological effect on the synovial cytokine profile cannot be ruled out. Finally, given that the majority of our patients were white, our results must be interpreted carefully and must be confirmed in other populations with different racial/ethnic distributions.

## Conclusions

We found no significant differences in synovial cell infiltrates nor in LN according to the ACPA status in our cohort of patients with RA. However, ACPA positive patients had higher levels of the IL-17 pathway-related cytokines/chemokines. Given that there were no differences between ACPA positive and negative patients with respect to systemic or local inflammation, these results suggest different pathogenic mechanisms in ACPA positive and negative RA patients.

## Abbreviations

ACPA: Anti citrullinated peptide/protein antibodies; CCL20: CC chemokine ligand 20; CCP2: Commercial anti-cyclic citrullinated peptide antibodies; CFFCP: Chimeric fibrin/filaggrin citrullinated synthetic peptides; CRP: C-reactive protein; DAS28: Disease activity score in 28 joints; DMARDs: Disease modifying anti-rheumatic drugs; ELISA: Enzyme-linked immunosorbent assay; ESR: Erythrocyte sedimentation rate; IL: Interleukin; IQR: Interquartile range; LN: Lymphoid neogenesis; mHAQ: modified health assessment questionnaire; PsA: Psoriatic arthritis; RA: Rheumatoid arthritis; RF: Rheumatoid factor; SF: Synovial fluid; SJC: Swelling joint count; SpA: Spondyloarthropathy; ST: Synovial tissue; TGF: Transforming growth factor; TJC: Tender joint count; TNF: Tumor necrosis factor.

## Competing interests

The authors declare they have no competing interests.

## Authors’ contributions

JAGP: study design, clinical and laboratory data collection, analysis and interpretation of data, wrote manuscript; RC: collection and analysis of data, drafting the figures; MVH, VRE and JR: clinical and laboratory data collection; IH: autoantibodies determination and analysis of data; JDC and RS: study design, analysis, interpretation of data and wrote manuscript. All authors read and approved the final manuscript.

## Supplementary Material

Additional file 1: Table S1 Immunohistochemistry characteristics quantified by Digital Image Analysis according to CCP titers (CCP2 high titers versus ACPA negative). Click here for file
