# Supplementary material for: Differences in synovial fluid cytokine levels but not in synovial tissue cell infiltrate between anti-citrullinated peptide/protein antibody-positive and –negative rheumatoid arthritis patients
Source: Arthritis Res Ther. 2013 Nov 7;15(6):R182. doi: 10.1186/ar4372 (PMC3978623; doi:10.1186/ar4372)
Supplement: Additional file 1: Table S1 — Immunohistochemistry characteristics quantified by Digital Image Analysis according to CCP titers (CCP2 high titers versus ACPA negative). [file ar4372-S1.docx]

**Supplementary Table 1. Immunohistochemistry characteristics quantified by Digital Image Analysis according to CCP titers (CCP2 high titers vs ACPA negative)**

|  | **Total**  **N=83** | **CCP positive**  **High titers (>1600 IU)**  **N=14** | **ACPA negative**  **N=19** | **p value** |
| --- | --- | --- | --- | --- |
| **CD3/mm²**  **(Median IQR 25-75)** | 509.9  (210.7-821.6)  n=70 | 447.2  (205.4 -629.2)  n=12 | 509.31 (233.8-898.2)  n=16 | 0.371 |
| **CD20/mm²**  **(Median IQR 25-75)** | 126.5  (30.3-242.6)  n=70 | 102.4  (54.7-265.4)  n=12 | 162.73 (42.2-315.0)  n=16 | 0.631 |
| **CD20-cell grade 3 follicles**  **(Median IQR 25-75)** | 0  (0-2.7)  n= 72 | 0  (0-1.7)  n=12 | 2.0  (0-3.5)  n=17 | 0.107 |
| **CD68L/mm²**  **(Median IQR 25-75)** | 293.3  (94.2-453.4)  n=63 | 326.7  (94.3-376.3)  n=8 | 204.74 (93.5-532.2)  n=16 | 1.0 |
| **CD68SL/mm²**  **(Median IQR 25-75)** | 677.1  (3.1-1665.5)  n=63 | 979  (240.3-2379.9)  n=8 | 526.74  (271.5-874.9)  n=16 | 0.264 |
| **CD15/mm²**  **(Median IQR 25-75)** | 58.4  (10-204.4)  n=49 | 71.9  (32.2-128.3)  n=5 | 35.74  (9.9-88.0)  n=14 | 0.444 |
| **CD117/mm²**  **(Median IQR 25-75)** | 40.2  (20.8-55.0)  n=51 | 39.15  (21-56.3)  n=5 | 42.81  (19.1-75.6)  n=14 | 0.622 |
| **CD31/34**  **(Median IQR 25-75)** | 46.3  (37.4-64.1)  n=44 | 68.0  (49.7-73.8)  n=5 | 43.56  (34.0-63.0)  n=13 | 0.059 |
| **LN (%)** | 42.5  n=73 | 25.0  n=12 | 58.8  n=17 | 0.71 |

Data are expressed as median (IQR) or as percentage.
